# Residual Cdk1/2 activity after DNA damage promotes senescence

**DOI:** 10.1111/acel.12588

**Published:** 2017-03-26

**Authors:** Erik Müllers, Helena Silva Cascales, Kamila Burdova, Libor Macurek, Arne Lindqvist

**Affiliations:** ^1^ Department of Cell and Molecular BiologyKarolinska InstitutetStockholmSweden; ^2^ Laboratory of Cancer Cell BiologyInstitute of Molecular GeneticsAcademy of Sciences of the Czech RepublicPragueCzech Republic; ^3^ Present address: Discovery SciencesAstraZeneca R&DGothenburg, MölndalSweden

**Keywords:** Cdk1, Cdk2, cell cycle, checkpoint recovery, DNA damage response, G2 phase, p21, senescence

## Abstract

In response to DNA damage, a cell can be forced to permanently exit the cell cycle and become senescent. Senescence provides an early barrier against tumor development by preventing proliferation of cells with damaged DNA. By studying single cells, we show that Cdk activity persists after DNA damage until terminal cell cycle exit. This low level of Cdk activity not only allows cell cycle progression, but also promotes cell cycle exit at a decision point in G2 phase. We find that residual Cdk1/2 activity is required for efficient p21 production, allowing for nuclear sequestration of Cyclin B1, subsequent APC/C^C^ ^dh1^‐dependent degradation of mitotic inducers and induction of senescence. We suggest that the same activity that triggers mitosis in an unperturbed cell cycle enforces senescence in the presence of DNA damage, ensuring a robust response when most needed.

## Introduction

In response to DNA damage, the cell cycle is halted to allow DNA repair. This is particularly critical in G2 phase, as entry into mitosis with unrepaired DNA may result in chromosomal aberration and propagation of mutations. However, upon DNA damage in S or G2 phase, the production of mitosis‐inducing factors such as Cyclin A2, Cyclin B1, Aurora A, Aurora B and Plk1 initially continues, albeit at a reduced pace (Bassermann *et al*., 2008; Wiebusch & Hagemeier, 2010; Müllers *et al*., 2014). As multiple feedback loops ensure a spiraling activation of Cyclin B1‐Cdk1, Cyclin A2‐Cdk1/2 and Plk1 that ultimately results in mitotic entry (Lindqvist *et al*., 2009b), maintaining even low levels of mitosis‐inducing factors poses the risk to eventually overrun a cell cycle arrest. In fact, suppression of Cdk activity merely by post‐translational modifications is insufficient to sustain a G2/M arrest (Jin *et al*., 1996, 1998; Bunz *et al*., 1998).

To avoid override of a cell cycle arrest, cells have evolved mechanisms that force terminal cell cycle exit and senescence. We and others have shown that terminal cell cycle exit from G2 phase depends on p53, its transcriptional target p21, and activation of the ubiquitin ligase APC/C^Cdh1^ that targets a large number of cell cycle regulators for degradation (Baus *et al*., 2003; Wiebusch & Hagemeier, 2010; Krenning *et al*., 2014; Müllers *et al*., 2014; Gire & Dulić, 2015). During this process, cells lose the expression of G2‐specific proteins, exit the cell cycle, and become senescent, thereby preventing propagation of mutations (Gillis *et al*., 2009; Nakayama & Yamaguchi, 2013; Johmura *et al*., 2014; Krenning *et al*., 2014; Gire & Dulić, 2015). What determines whether and when a cell in G2 phase becomes senescent remains unclear.

There are several indications that induction of senescence is a regulated process. Terminal cell cycle exit is a sharp transition, whose point‐of‐no‐return is marked by the translocation of Cyclin B1 from the cytoplasm to the nucleus. The translocation of Cyclin B1 requires p21, which presumably sequesters Cyclin B1/Cdk1 in the nucleus (Krenning & Medema, 2014; Krenning *et al*., 2014; Müllers *et al*., 2014). Before terminal cell cycle exit is initiated in G2 phase, there is a variable delay, whose duration depends on when within S or G2 phase the damage occurred. That is, a cell receiving damage in late G2 exits the cell cycle faster than a cell receiving damage in early G2 phase (Müllers *et al*., 2014). These observations suggest that the signaling pathways that mediate senescence and cell cycle progression are interlinked.

Here we show that, despite a severe suppression, Cdk activity persists during a cell cycle arrest until terminal cell cycle exit occurs and that this remaining Cdk activity stimulates senescence. We suggest that the key activity that drives mitosis in the absence of DNA damage promotes cell cycle exit in the presence of DNA damage.

## Results

### Concerted Cdk1 and Cdk2 activity regulates Cyclin B1 nuclear accumulation and induction of senescence upon DNA damage

We previously employed live‐cell microscopy of individual RPE cells encoding a Cyclin B1‐eYFP fusion protein at the endogenous *CCNB1* locus to study terminal cell cycle exit. Using this system, we observed that DNA damage‐dependent nuclear accumulation and degradation of Cyclin B1 occurred only after S‐phase completion (Müllers *et al*., 2014). As this is the time when Plk1 and Cdk1 are activated in an unperturbed cell cycle (Akopyan *et al*., 2014), we hypothesized that cell cycle kinases might be involved in regulating nuclear accumulation and degradation of Cyclin B1 after DNA damage. To test this idea, we monitored the effect of different cell cycle kinase inhibitors on Cyclin B1‐eYFP levels and localization after DNA damage using time‐lapse microscopy.

**Figure 1:**
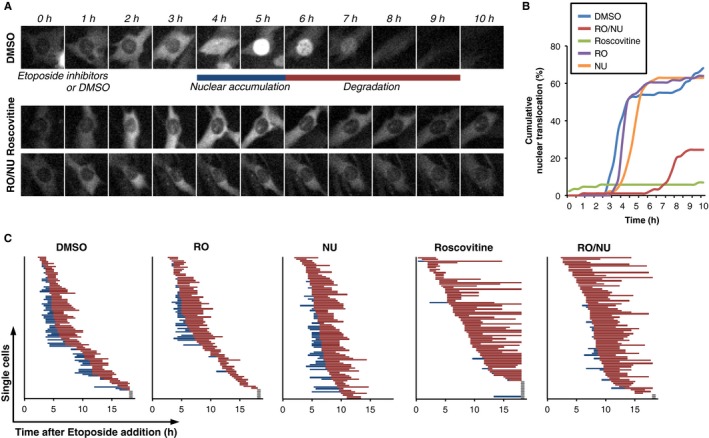
Concerted Cdk1 and Cdk2 activity regulates Cyclin B1 nuclear accumulation upon DNA damage. RPE Cyclin B1‐eYFP cells were treated with Etoposide and followed by time‐lapse microscopy. After 1 h cells were treated with MG‐132, Roscovitine, RO‐3306, NU6140, inhibitor combinations as indicated, or mock treated with DMSO (Control). (A) Representative images of single cells. (B) Cumulative nuclear accumulation of Cyclin B1‐eYFP was assessed in more than 150 cells for each condition. (C) The time point of nuclear accumulation and the onset of degradation of Cyclin B1‐eYFP were determined in single cells, as outlined in (A). Each line represents a single cell. Blue lines indicate predominantly nuclear Cyclin B1, red lines indicate degradation of Cyclin B1, and gray lines indicate cells with no detectable Cyclin B1 degradation.

We used continuous exposure to the topoisomerase II inhibitor Etoposide, which efficiently induces Cyclin B1‐eYFP nuclear accumulation and degradation in G2 phase (Müllers *et al*., 2014). To avoid possible interference with initiation of a DDR, we added kinase inhibitors 1 h after addition of Etoposide. Addition of a potent inhibitor of Plk1 (BI2536) did not affect Cyclin B1‐eYFP degradation or intracellular localization upon DNA damage, indicating that Plk1 is not required for these events (Fig. S1A, Supporting information). On the contrary, combined addition of selective Cdk1 (RO‐3306) and Cdk2 (NU6140) inhibitors, or addition of the broad Cdk inhibitor Roscovitine almost completely abolished nuclear localization of Cyclin B1‐eYFP and delayed the onset and duration of Cyclin B1‐eYFP degradation (Figs 1A–C and S1B–E, Supporting information). Similarly, stimulation of cellular Cdk activity by inhibition of Wee1 (MK1775) led to earlier degradation along with higher rates of DNA damage checkpoint slippage (Fig. S1F, Supporting information). Taken together, our results suggest that Cdk1/2 activity is needed for timely Cyclin B1 translocation and degradation after DNA damage.

**Figure 2:**
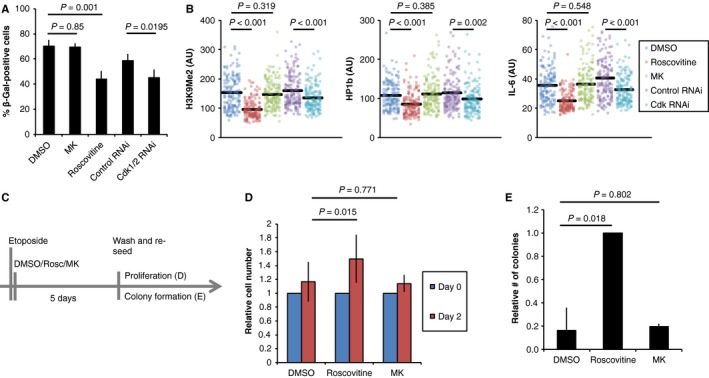
Cdk activity promotes senescence induction upon DNA damage. (A) Mean and standard deviation from four independent experiments of RPE cells treated with Etoposide and after 1 h with Roscovitine, MK‐1775 (MK) or with DMSO. Alternatively cells were transfected with siRNA for Cdk1 and Cdk2 at both 24 and 48 h before damage induction in three independent experiments. Cells were stained for β‐Galactosidase 4 days later. Statistical hypothesis testing was performed using two‐sided *t*‐test. (B) Quantification of nuclear H3K9Me2, HP1b, and IL‐6 levels in RPE cells treated with Etoposide and after 1 h with Roscovitine, MK‐1775 (MK) or with DMSO. Alternatively cells were transfected with siRNA for Cdk1 and Cdk2 at both 24 and 48 h before damage induction. Cells were fixed 5 days after damage induction. Statistical hypothesis testing was performed using two‐sided *t*‐test. (C) Schematic of setup used for D, E. (D) Analysis of proliferative capacity. RPE cells were treated with Etoposide and 1 h later with Roscovitine, MK‐1775 or DMSO. Inhibitors were kept on for 5 days, then cells were counted, reseeded into fresh medium without Etoposide or kinase inhibitors and counted again after 2 more days. Experiments were run in quadruplicates. Mean and standard deviation of three independent experiments are shown. Statistical hypothesis testing was performed using two‐sided *t*‐test. (E) Analysis of clonogenic capacity. RPE cells were treated with Etoposide and 1 h later with Roscovitine, MK‐1775, or DMSO. Inhibitors were kept on for 5 days, and then, 5000 cells were reseeded into fresh medium without Etoposide or kinase inhibitors and the number of colonies was assessed 1 week later. Experiments were run in quadruplicates. Normalized mean and standard deviation of three independent experiments are shown. Statistical hypothesis testing was performed using two‐sided *t*‐test.

DNA damage‐induced nuclear accumulation of Cyclin B1 in G2 phase marks a decision point for terminal cell cycle exit and senescence (Krenning *et al*., 2014; Müllers *et al*., 2014). As inhibition of Cdk1/2 activity substantially delayed nuclear accumulation of Cyclin B1, we next sought to test if Cdk activity affects whether cells become senescent. To this end, we assessed senescence‐associated markers while perturbing Cdk activity using kinase inhibitors or Cdk RNAi. We quantified the occurrence of β‐Galactosidase staining (Figs 2A and S2A, Supporting information), total and foci‐associated staining of H3K9Me2 and HP1b as markers of senescence‐associated heterochromatin foci (SAHF), as well as expression of IL‐6 as marker for the senescence‐associated secretory phenotype (SASP) (Figs 2B and S2B–E, Supporting information). While long‐term treatment with RO‐3306 and NU6140 was toxic for cells, we found that all these markers were reduced upon combined Cdk1/2 RNAi or addition of Roscovitine, indicating that Cdk activity stimulates senescence. Consistent with previous reports, addition of Roscovitine led to a reduction in the amount of viable cells. Importantly, combined treatment with Roscovitine and Etoposide did not further decrease the amount of viable cells (Fig. S2F,G, Supporting information). Further, the levels of DNA damage markers were not changed by combining Etoposide with Roscovitine compared to Etoposide alone, indicating that Roscovitine did not cause additional DNA damage (Fig. S4B,C, Supporting information). The proliferation capacity, measured by total cell numbers and clonogenic growth, increased after temporal Cdk inhibition (Figs 2C–E and S2G, Supporting information). Increased Cdk activity may however not necessarily lead to increased senescence, as we find no evidence that Wee1 inhibition changed senescence‐associated markers or proliferation capacity during constant exposure to Etoposide for 4 or 5 days (Figs 2A–E and S2E,H, Supporting information). Thus, our data suggest that Cdk activity after DNA damage stimulates induction of senescence, but also that cells contain sufficient Cdk activity to promote senescence during prolonged exposure to a DNA‐damaging compound.

### Low levels of Cdk activity are preserved for several hours during a DDR in a cell cycle‐dependent manner

**Figure 3:**
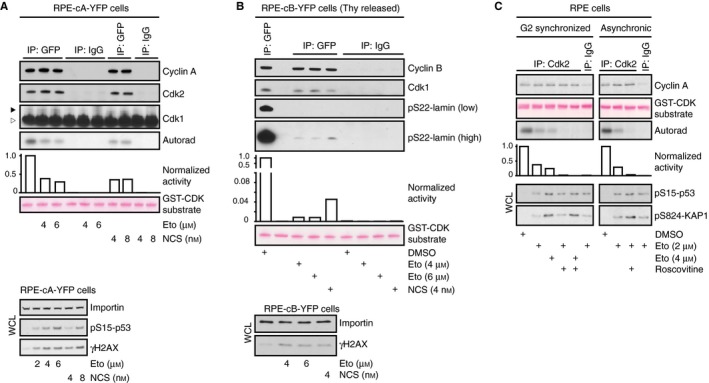
Cyclin A‐ and Cyclin B‐associated Cdk activity is preserved upon DNA damage. (A) Asynchronous growing RPE Cyclin A2‐eYFP cells were treated with Etoposide or NCS for 4 h, lysed and immunoprecipitated with anti‐GFP or control antibody. Kinase assay was performed using GST‐Cdk substrate peptide, and phosphorylation was detected by autoradiography. Co‐immunoprecipitation of Cdk2 and Cdk1 was determined by immunoblotting. Arrowhead shows position of Cdk1, empty arrowhead indicates position of IgG. WCL, whole cell lysate. (B) RPE Cyclin B1‐eYFP cells were released for 6 h from a thymidine block, treated with Etoposide or NCS for 4 h, lysed and immunoprecipitated with anti‐GFP or control antibody. Kinase assay was performed using GST‐LAMS22 substrate peptide, and phosphorylation was detected by antibody against Lamin A/C phosphorylated at Ser22. WCL, whole cell lysate. (C) RPE cells were released for 6 h from a thymidine block, treated with Etoposide for 4 h, lysed and immunoprecipitated with anti‐Cdk2 or control antibody. Kinase assay was performed in the absence or presence of Roscovitine, and kinase activity was determined as in (a).

Although a DNA damage‐mediated checkpoint largely functions by blocking Cdk activity, our data and that of others (Cerqueira *et al*., 2009) indicate that Cdk activity is integrated into the cellular response to DNA damage. To resolve this apparent paradox, we sought to assess whether and to which extent Cdk activity can persist when challenged by high levels of DNA damage. To assess Cdk activity *in vitro,* we immunoprecipitated Cyclin A2‐eYFP or Cyclin B1‐eYFP from gene‐targeted RPE cells (Akopyan *et al*., 2014; Müllers *et al*., 2014) and performed kinase assays on recombinant target proteins that can be phosphorylated by either Cdk2 or Cdk1. Cyclin A2‐associated Cdk is active through a large part of interphase (Hochegger *et al*., 2008) and was readily detected in a population of unsynchronized cells. Although significantly reduced, Cyclin A2‐eYFP‐associated activity persisted 4 h after addition of either Etoposide or the radiomimetic drug Neocarzinostatin (NCS) (Fig. 3A). In contrast, Cyclin B1‐associated Cdk activity is initially activated at the S/G2 border and slowly builds up through G2 phase until a dramatic increase initiates mitosis (Akopyan *et al*., 2014). Indeed, we detect a strong Cyclin B1‐eYFP‐associated kinase activity in RPE cells at 10 h after release from a thymidine block. Even though markedly reduced, a very low level of Cyclin B1‐eYFP‐associated kinase activity was still detectable after 4‐h Etoposide or NCS treatment, when no mitotic cells were visually detected (Fig. 3B). This indicates that although low compared to the activities that initiate mitosis, both Cyclin A‐ and Cyclin B‐associated activities are present after DNA damage. Similarly, immunoprecipitated Cdk2 from both unsynchronized and G2 synchronized RPE cells showed reduced but persistent activity 4 h after Etoposide treatment (Fig. 3C). Thus, Cdk activity persists at a low level after DNA damage in RPE cells.

**Figure 4:**
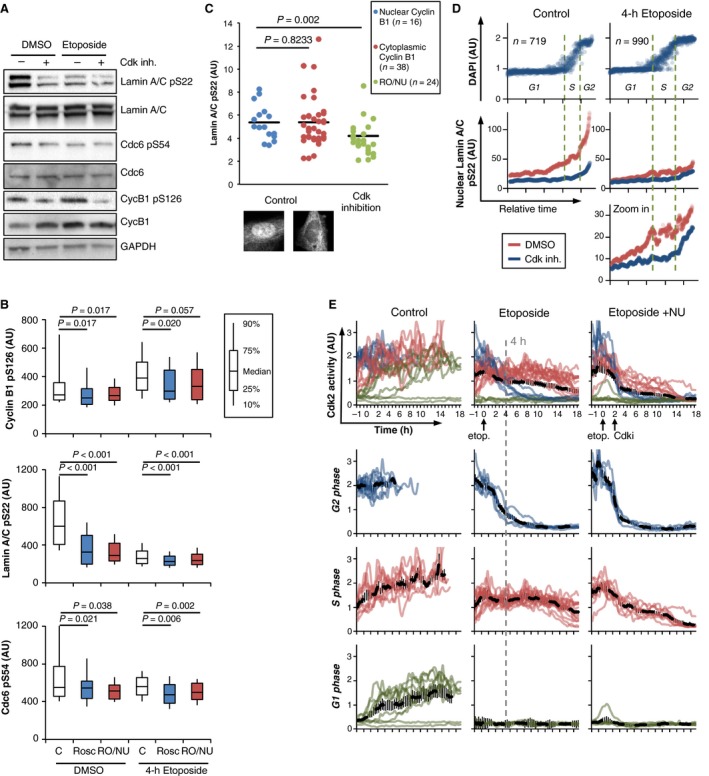
Low levels of Cdk activity are preserved for several hours upon DNA damage in a cell cycle‐dependent manner. (A) Representative Western blot of RPE cells treated with Etoposide or mock treated with DMSO and harvested after 4 h. Cells were treated with DMSO (Control) or a combination of Roscovitine, RO‐3306 and NU6140 (Cdk inh.) 1 h before harvesting. (B) Immunofluorescence quantification of Cyclin B1 pS126, Lamin A/C pS22, and Cdc6 pS54 nuclear fluorescence intensity of interphase RPE cells with 4n DNA content. Cells were treated with Etoposide or mock treated with DMSO and fixed after 4 h. Cdk inhibitors were added 1 h before fixation. More than 250 cells were analyzed for each condition. G2 cells were identified from DAPI staining. Statistical hypothesis testing was performed using two‐sided *t*‐test. (C) Quantification of nuclear Lamin A/C pS22 in single G2 RPE cells. Cells were treated as in (B). Cells in G2 phase were identified according to DNA content using DAPI staining. The images show representative single cells with predominantly nuclear or cytoplasmic Cyclin B1. (D) Quantification of DNA content (DAPI) and nuclear Lamin A/C pS22 vs. estimated time in RPE cells. Cells were sorted for DAPI and Cyclin B1. The Lamin A/C pS22 quantifications show a running median of 41 cells. The lower panel shows a zoom‐in of the middle panel. Cells were treated with Etoposide or mock treated with DMSO (Control) and fixed after 4 h. One hour before fixation, cells were treated with a combination of Roscovitine, RO‐3306 and NU6140, or with DMSO. (E) Quantification of CDK2 activity in individual, live cells in control and damage conditions. RPE cells expressing a Cdk2 activity probe (Spencer *et al*., 2013) were followed up to entry into mitosis (Control). The traces were color‐coded according to initially low (green), intermediate (red), or high (blue) Cdk2 activity, indicating cells in G1 phase, S phase, or G2 phase, respectively (Spencer *et al*., 2013). Dashed black lines indicate the respective average. The dashed gray line serves as an indicator of the 4‐h time point analyzed in fixed‐cell experiments.

To assess Cdk activity *in vivo,* we next studied phosphorylation of endogenous Cdk targets in damaged and unperturbed RPE and U2OS cells. As the total phosphorylation levels provide little information on when phosphorylation occurred and whether a kinase is continuously active, we added Cdk inhibitors during the last hour of a 4‐h Etoposide treatment. For both cell lines, addition of Cdk inhibitors after Etoposide treatment reduced Cdk target phosphorylation in whole cell populations (Figs 4A and S3A–D, Supporting information) as well as in single G2 cells (Figs 4B and S3E, Supporting information). Thus, our data suggest that Cdk activity is present between 3 and 4 h after Etoposide addition.

To assess how long Cdk activity is sustained, we next sought to investigate Cdk target phosphorylation in individual G2 cells and use Cyclin B1 presence and localization as a marker for cell cycle exit. We find that Cdk target phosphorylation is still detectable in cells with mainly nuclear Cyclin B1 after 4 h of Etoposide treatment, but not after 24 h when Cyclin B1 is degraded. This suggests that full Cdk inactivation occurs only after Cyclin B1 nuclear accumulation and that Cdk activity persists until cell cycle exit (Figs 4C and S3F, Supporting information). Notably, Cdk target phosphorylation correlated positively with the levels of the DNA damage marker ɣH2AX, thus excluding the possibility that only mildly damaged cells retain Cdk activity (Fig. S3G, Supporting information). Furthermore, ɣH2AX levels were not affected by RO/NU treatment showing that Cdk inhibition does not result in an overall reduction in DNA damage signaling (Fig. S3G, Supporting information). To assess the cell cycle distribution of Cdk activity after DNA damage, we performed quantitative immunofluorescence for Cdk‐dependent phosphorylation and sorted the cells according to their relative position in the cell cycle (Akopyan *et al*., 2014, 2016; Müllers *et al*., 2014). To detect ongoing Cdk activity and to control for cell cycle‐dependent differences in background signals and target site specificity, we added Cdk inhibitors 1 h before fixation. In accordance with recent data on Cdk2 (Spencer *et al*., 2013), we detected initial Cdk1/2 target phosphorylation already during G1, from where it slowly rose throughout S‐phase before it rapidly increased at the S/G2 border (Fig. 4D, ‘Control’). Strikingly, this cell cycle‐dependent pattern of Cdk target phosphorylation was still preserved after 4 h of continuous Etoposide treatment, albeit at a lower level (Fig. 4D, ‘4‐h Etoposide’). Inhibition of Cdk1 and Cdk2 decreased Lamin A/C phosphorylation synergistically throughout interphase, indicating a redundancy between both kinases (Fig. 4D, ‘4‐h Etoposide’; and Fig. S3H, Supporting information). In summary, we detect Cdk‐dependent target phosphorylations in single cells throughout all cell cycle phases after DNA damage.

We next made use of a Cdk2 activity sensor (Spencer *et al*., 2013) to obtain an independent readout of Cdk2 activity in individual living cells and to gain further insights into the dynamics of Cdk activity during a DDR. In cells with initial high levels of Cdk2 activity, presumably in G2 phase, full inhibition was reached after 6–8 h of constant Etoposide treatment (Fig. 4E, blue tracks). In contrast, cells with initial intermediate levels of Cdk2 activity (suggestive of a S‐phase state) sustained this level of Cdk activity for 16 h and more (Fig. 4E, red tracks). This result is in line with our previous observation that S‐phase cells slowly progress to G2 phase before exiting the cell cycle (Müllers *et al*., 2014).

Taken together, our data show that low levels of Cdk activity persist even upon DNA damage sufficient to cause cell cycle exit in a vast majority of cells. The duration and extent of sustained Cdk activity depends on the cell cycle position when DNA damage occurred.

### Cdk activity during DNA damage promotes p21 production

**Figure 5:**
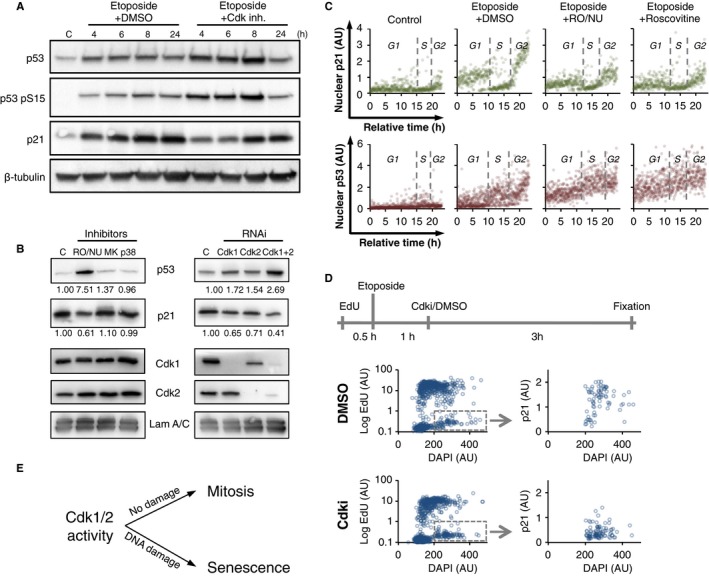
Cdk activity during DNA damage promotes p21 production. (A) Representative Western blot of RPE cells treated with Etoposide and with a combination of Roscovitine, RO‐3306 and NU6140 (Cdk inh.), or mock treatment with DMSO 1 h later. Cell lysates were prepared at the indicated time points. C, control. (B) Representative Western blots of RPE cells treated with Etoposide and with Roscovitine (Cdk1/2), MK1775 (Wee1), SB202190 (p38), or mock treatment with DMSO 1 h later (left blot). Alternatively cells were transfected with the indicated siRNA at 24 and 48 h before damage induction (right blot). C, Control. (C) Quantification of nuclear p21 levels and nuclear p53 level vs. estimated time. Cells were sorted for DAPI and Cyclin B1. Cells were treated with Etoposide at 1 μm concentration or mock treated with DMSO (control). Roscovitine, a combination of RO‐3306 and NU6140, or DMSO was added 1 h after Etoposide treatment. Cells were fixed after 4 h. More than 350 cells were analyzed for each condition. (D) Quantification of p21 levels in cells that were in G2 phase upon Etoposide addition. EdU, Etoposide, and a combination of RO‐3306 and NU6140 were added to cells with 30‐ and 60‐min intervals, according to schematic; 3 h later, cells were fixed and analyzed by microscopy. Left graphs show gate of EdU negative 4N DNA content cells, representing cells that were in G2 phase upon addition of Etoposide. Right graphs show p21 levels of gated cells. *N* = 2. (E) Cdk activity determines cell fate decisions toward mitosis in unperturbed conditions or toward senescence upon DNA damage.

Cell cycle exit and senescence from G2 phase after DNA damage depend on p53 and its transcriptional target p21 (Niculescu *et al*., 1998; Charrier‐Savournin *et al*., 2004; Wiebusch & Hagemeier, 2010; Müllers *et al*., 2014). We therefore sought to investigate whether Cdk activity could enhance p53 and p21 expression. In contrast to this hypothesis, we found p53 expression to be elevated when Cdk1 and Cdk2 were inhibited or knocked down, suggesting that Cdk activity does not enhance p53 levels (Figs 5A,B and S4A, Supporting information). Strikingly however, despite an increase in p53, p21 induction was reduced upon Cdk inhibition or depletion, suggesting that the remaining Cdk activity during a DDR promotes p21 expression (Figs 5A,B and S4A,D, Supporting information). Further, expression of a constitutively active Cdk1‐AF mutant leads to increased p21 levels, supporting a role for Cdk activity to promote p21 expression (Fig. S4E, Supporting information). Our data thus suggest that blocking Cdk activity during DNA damage results in an increase in p53 expression, but a decrease in p21 expression. These data are somewhat surprising, given that p21 is a well‐established transcriptional target of p53 (el‐Deiry *et al*., 1993).

To further investigate Cdk‐dependent p21 induction, we next focused on the determinants of p21 expression. Although p21 was induced by Etoposide addition, p21 levels in single cells correlated poorly with the levels of ɣH2AX staining, indicating that p21 expression is not solely regulated by the amplitude of DNA damage signaling present in a cell (Fig. S4C, Supporting information). Instead, the extent of p21 induction upon Etoposide treatment correlates strongly to cell cycle stage: p21 was expressed in all cells in G1 phase, virtually absent in cells in S‐phase (Abbas *et al*., 2008), and dramatically increased in cells that had crossed the S/G2 border (Fig 5C). To investigate whether combined addition of Etoposide and Cdk1/2 inhibition decreased p21 levels by delaying passage through S‐phase, we specifically monitored cells that were in G2 phase upon Etoposide addition. Cdk1/2 inhibition reduced p21 levels in G2 cells, indicating that reduced expression of p21 after Cdk1/2 inhibition can occur after completion of S‐phase (Figs 5D and S5A,B, Supporting information). Similarly, inhibition of Cdk1 and Cdk2 significantly decreased p21 induction in G1 as well a G2 phase cells, indicating that Cdk activity affects p21 expression throughout the cell cycle (Fig. 5C, top panel). Analyzing p53 levels, we found stronger induction upon DNA damage in S‐phase and G2‐phase compared to G1‐phase cells. When Cdk activity was inhibited, p53 levels were elevated in all cell cycle phases, again reaching the highest expression in S and G2 phase cells (Fig. 5C bottom panel). Thus, our data indicate that the expression levels of p53 and p21 are differentially regulated upon DNA damage and that Cdk activity is needed to induce high p21 levels.

We next sought to test whether Cdk activity affects production or degradation of p21. Addition of the proteasome inhibitor MG‐132 and the protein translation inhibitor Cycloheximide affected p21 levels in Etoposide‐treated G2 cells, suggesting that p21 is continuously produced and degraded. Combined Cdk inhibition and proteasome inhibition resulted in lower p21 levels than in control Etoposide‐treated cells, suggesting that Cdk‐mediated p21 expression cannot be explained solely by differences in p21 degradation (Fig. S5C, Supporting information). In line with this finding, Cdk activity did not significantly affect p21 stability after DNA damage (Fig. S5D, Supporting information). In contrast, we detect a 20–30% decrease in p21 mRNA levels after Cdk inhibition in Etoposide‐treated cells (Control) (Fig. S5E,F, Supporting information). Thus, we find that upon DNA damage, Cdk activity enhances p21 protein levels. Although the relatively low reduction in p21 mRNA levels after Cdk inhibition indicates that additional mechanisms exist, our data suggest that Cdk activity contributes to p21 expression in part by increasing the amount of p21 mRNA.

## Discussion

A paradox in the cellular response to DNA damage is that a checkpoint is enforced by inhibiting Cdk activity, whereas numerous reports implicate that Cdk activity is needed in a DNA damage response for DNA replication, homologous recombination, and DNA repair (Jazayeri *et al*., 2006; Myers *et al*., 2007; Cerqueira *et al*., 2009; Johnson *et al*., 2009; Tian *et al*., 2009; Ferretti *et al*., 2013). Here we show that although the DDR‐mediated inhibition of Cdk activity is efficient, low levels of Cdk activity persist until terminal cell cycle exit. We suggest that these levels are sufficient to continue Cdk‐dependent cell cycle functions such as DNA replication (Bartek *et al*., 2004) and accumulation of mitotic cyclins (Fung & Poon, 2005; Müllers *et al*., 2014), as well as to promote DNA repair (Yata & Esashi, 2009), and to maintain the cellular competence for checkpoint recovery (Lindqvist *et al*., 2009a; Alvarez‐Fernandez *et al*., 2010). Maintaining low levels of Cdk activity during a DDR could provide a necessary time window of slow cell cycle progression in which repair and eventual recovery is possible.

While the remaining Cdk activity sustains important cellular functions during a DDR, it also poses a risk for genome stability. If cells with damaged DNA progress into G2 phase, they need to be prevented from entering mitosis, which otherwise could result in chromosome missegregation and propagation of mutations. Indeed, in the absence of p53 or p21, a cell cycle arrest in G2 phase is eventually overrun (Bunz *et al*., 1998). We show that Cdk activity is coupled to negative feedback by inducing p21 expression. P21 can bind and inhibit Cyclin/Cdk complexes, and is required for sequestration of Cyclin B1 in the nucleus and terminal cell cycle exit (Baus *et al*., 2003; Wiebusch & Hagemeier, 2010; Krenning *et al*., 2014; Müllers *et al*., 2014; Gire & Dulić, 2015). As increasing Cdk activity drives mitotic entry, the incorporation of Cdk activity as a positive regulator of p21 expression provides an elegant mechanism to ensure cell cycle exit and senescence when most needed. Thus, our data highlight the overall importance of Cdk activity as cellular read‐out for cell cycle position (Stern & Nurse, 1996; Uhlmann *et al*., 2011) and as a regulator of key cell fate decisions (Fig. 5E).

We find that inhibition of Cdk1/2 activity after Etoposide treatment results in reduction of p21 mRNA levels. Given the crucial role of p21 in promoting senescence from G2 phase (Gire & Dulić, 2015), we suggest that Cdk1/2 activity at least in part promotes senescence by enhancing the levels of p21 mRNA. Whether p21 mRNA levels are increased due to enhanced transcription or stability remains unclear. However, we note that similar to p21 levels, Chk1 activity is reduced after Cdk1/2 inhibition in Etoposide‐treated cells (Fig. S4B, Supporting information). Decreased activation of ATR/Chk1 is likely caused by decreased resection of DNA ends, which requires Cdk activity (Jazayeri *et al*., 2006; Ferretti *et al*., 2013). As Chk1 can phosphorylate p53 (Ou *et al*., 2005), it will be interesting to investigate whether Cdk1/2 can enhance p21 mRNA levels through Chk1.

We detect decreased p21 levels and delayed Cyclin B translocation within hours after Cdk1 inhibition of Etoposide‐treated cells. Correspondingly, after prolonged exposure to Etoposide and Roscovitine, we detect reduced expression of senescence markers. However, we note that albeit reduced, senescence markers accumulate also after several days of combined treatment of Etoposide and Roscovitine. This suggests that while Cdk activity promotes timely onset of senescence, it may not be essential for cells to eventually become senescent. In addition, long‐term treatment with Cdk inhibitors may have pleiotropic effects, and we do detect reduced metabolic activity and apoptosis after prolonged exposure to Roscovitine.

The acquisition and maintenance of a G2 DNA damage arrest is very different in transformed and untransformed cells, largely due to misregulation of p53 and p21. This provides both chances and challenges for the selective treatment of cancer cells. Cdk inhibitors in general and Roscovitine (Seliciclib) in particular have shown promising results as part of a combined cancer therapy (Lapenna & Giordano, 2009; Johnson *et al*., 2011). The antitumor activity and specificity of Cdk inhibitors is mainly attributed to the increased requirement of Cdk activity in highly proliferating tumor cells. In addition, Cdk inhibitors have been proposed as drug candidates in combination with radiation therapy or chemotherapy (Maggiorella *et al*., 2003). Our results raise some caution as they suggest that inhibition of Cdk activity during DNA damage interferes with checkpoint signaling, namely p21 expression and induction of senescence. This could lead to increased checkpoint override in the presence of DNA damage and potentially to sustained proliferation of damaged nontumor cells.

We propose that upon DNA damage, a low level of cell cycle‐dependent Cdk activity is retained. This activity exerts an important dual role within the DNA damage checkpoint response. In S‐phase, Cdk activity allows cells to continue cell cycle progression, DNA replication, and repair. In G2 phase, Cdk activity promotes p21 production, creating a negative feedback loop that constitutes a logical gate to ensure a robust decision toward terminal cell cycle exit. Interestingly, mutations in oncogenes frequently result in increased Cdk activity, thereby driving cell proliferation, and inducing DNA replication stress (Macheret & Halazonetis, 2015). Our data suggest that increased Cdk activity in combination with replication stress would lead to increased cell cycle exit from G2, which is in line with the robust senescence block that is observed as an early barrier for tumor progression (Halazonetis *et al*., 2008).

## Experimental procedures

### Cell culture

RPE and U2OS cell lines were cultured in an ambient‐controlled incubator at 37 °C with 5% CO_2_ in Dulbecco's modified eagle medium (DMEM)/Nutrient mixture F‐12 (DMEM/F‐12) + GlutaMAX (Invitrogen, Carlsbad, CA, USA) supplemented with 10% FBS (FBS, HyClone, South Logan, Utah, USA) and 1% Penicillin/Streptomycin (Pen/Strep; HyClone), and DMEM + GlutaMAX (Invitrogen) supplemented with 6% heat‐inactivated fetal bovine serum and 1% Pen/Strep, respectively. For live‐cell imaging experiments, cells were cultured in Leibowitz's L‐15 medium (Invitrogen) supplemented with 10% FBS and 1% Pen/Strep.

### Plasmids, cloning, purification, and transfection

The use of the live‐cell sensor for Cdk2 activity (kindly provided by Tobias Meyer and Sabrina Spencer) has been described previously (Spencer *et al*., 2013).

For experiments involving constitutively active Cdk1, cells were transfected using 2.5 μg Cdk1AF‐GFP (kindly provided by Rob Wolthuis) or ECFP1‐C1 (Clontech) using Lipofectamine 2000 (Invitrogen) 24 h before analysis of the phenotype.

To clone substrates for the kinase assays, DNA fragments corresponding to the optimal Cdk2 substrate peptide HHASPRK or STPLSPTRIT peptide derived from Lamin A were ligated in frame into pGEX6P plasmid (Brown *et al*., 1999). GST, GST‐CDK2, or GST‐LAMS22 substrates were purified from BL21 bacteria induced by 0.5 mm IPTG for 5 h using glutathione beads.

### Inhibitors and RNAi

The inhibitors used in this study were employed at the following concentrations: Roscovitine at 25 μm (Cdk inhibitor; Selleck Chemicals, Houston, TX, USA), NU6140 at 10 μm (Cdk2 inhibitor; Calbiochem), RO‐3306 at 10 μm (Cdk1 inhibitor; Calbiochem, Darmstadt, Germany), MG‐132 at 10 μm (Inhibitor of the proteasome; Sigma‐Aldrich, Saint Louis, Missouri, USA), BI2536 at 100 nm (Plk1 inhibitor; Selleck Chemicals), MK‐1775 at 100 μm (Wee1 inhibitor; Selleck Chemicals), Nutlin‐3 at 13 μm (Mdm2 antagonist; Sigma‐Aldrich), SB202190 at 10 μm (p38 inhibitor; Selleck Chemicals), Cycloheximide at 10 μg mL^−1^ (Inhibitor of protein translation; Sigma‐Aldrich). Etoposide (topoisomerase II inhibitor; Sigma‐Aldrich) was employed at 1 μm, which is sufficient to induce robust checkpoint arrest in cells at all cell cycle stages (Müllers *et al*., 2014). Figure S1D (Supporting information) shows the robustness of the checkpoint arrest as well as the efficiency of Cdk inhibitor treatment.

SMARTpool ON‐TARGET plus siRNAs targeting CDKN1A (p21), CDK1, or CDK2 were purchased from Dharmacon and employed at a concentration of 20 nm using HiPerFect (Qiagen, Hilden, Germany) transfection at 48 and 24 h before analysis of the phenotype.

### Live‐cell microscopy and quantitative immunofluorescence

Live‐cell imaging experiments were carried out as previously described (Müllers *et al*., 2014).

For quantitative immunofluorescence, cells were fixed and immunostained as previously described (Müllers *et al*., 2014). Images were acquired on an ImageXpress system (Molecular Devices, Sunnyvale, CA, USA) using a 40× NA 0.6 or a 60× NA 0.7 objective. Images were manually screened for aberrant staining or illumination, and processed and analyzed using CellProfiler and ImageJ. Background subtraction and image analysis for the identification of cell nuclei was essentially carried out as previously described (Macurek *et al*., 2013). Figure S3I (Supporting information) shows imaging examples and analysis including several controls. To assess kinetics from quantitative immunofluorescence, cells were ordered based on increasing, median‐normalized DAPI, and/or nuclear Cyclin B1 fluorescence (Akopyan *et al*., 2014, 2016). The cells were linearly distributed according to their fluorescence value between the time 0 and 23 h. The approximate borders between cell cycle phases were visually identified according to the DAPI profile (Akopyan *et al*., 2014, 2016). Foci analysis was performed by subtracting a Gaussian filter blurred image from the original image, and measuring the integrated intensity of the resulting foci per nucleus using cellprofiler.org. The following antibodies were used: Cyclin B1V152 (1:400; #4135 Cell Signaling, Danvers, MA, USA), ɣH2AX (1:400; #9781 Cell Signaling), p53 7F5 (1:200; #2527 Cell Signaling), p21 12D1 (1:400; #2947 Cell Signaling), Lamin A/C pS22 (1:400; #2026 Cell Signaling), Cyclin B1 pS126 (1:200; ab55184 Abcam, Cambridge, UK), Cdc6 pS54 EPR759Y (1:200; ab75809 Abcam), IL6 (1:1000; ab9324 Abcam), H3K9Me2 (1:500; ab1220 Abcam), HP1b 1MOD‐1A9 (1:1000; MAB3448 Millipore, Darmstadt, Germany).

### Immunoblotting

The following antibodies were used: Lamin A/C pS22 (1:400; #2026 Cell Signaling), Cdc6 pS54 EPR759Y (1:200; ab75809 Abcam), Cyclin B1 pS126 (1:200; ab55184 Abcam, 1:100; ab3488 Abcam), p53 DO‐1 (1:500; sc‐126 Santa Cruz, Dallas, TX, USA), p53 pSer15 (1:200, #9284 Cell Signaling), p21 12D1 (1:1000; #2947 Cell Signaling), β‐tubulin 9F3 (1:1000; #2128S Cell Signaling), Cdk1 POH1 (1:1000; #9116 Cell Signaling), Cdk1 (1:200; HPA003387 Atlas antibodies), Cdk2 78B2 (1:1000; #2564 Cell Signaling), Lamin A/C (1:2000; #4777 Cell Signaling), GAPDH (1:15000‐25000; G9545 Sigma‐Aldrich), pKap1 (1:500; A300‐767A Bethyl Antibodies, Montgomery, TX, USA), and pChk2 (1:1000; #2661 Cell Signaling).

### qPCR

For RT–qPCR, total RNA was extracted from the cells with an RNeasy Mini kit (Qiagen). Reverse transcriptase reaction was set up using 250 ng of total RNA and 10 pmol oligo (dT) primers. The qPCRs were set up using Fast SYBR Green Master Mix (life technologies). Samples were run on a 7500 Fast Real‐Time PCR System (life technologies) using the following primers: p21‐forward (5′‐AGG CAC CGA GGC ACT CAG AG‐3′), p21‐reverse (5‐AGT GGT AGA AAT CTG TCA TGC TG‐3), p53‐forward (5′‐ATG GAG GAG CCG CAG TCA GAT‐3′), p53‐reverse (5′‐GCA GCG CCT CAC AAC CTC CGT C‐3′), FOXM1‐forward (5′‐ACC CAA ACC AGC TAT GAT GC‐3′), FOXM1‐reverse (5′‐GAA GCC ACT GGA TGT TGG AT‐3′), GAPDH‐forward (5′‐CGG AGT CAA CGG ATT TGG TCG TAT‐3′), GAPDH‐reverse (5′‐AGC CTT CTC CAT GGT GGT GAA GAC‐3′). Primers were obtained from Sigma‐Aldrich.

### Senescence‐associated β‐Galactosidase assay, cell proliferation, and clonogenic assay

β‐Gal stainings were performed using the ‘Senescence β‐Galactosidase Staining Kit’ (Cell Signaling).

To determine cell proliferation potential, cells were seeded in quadruplicates in 6‐well plates 1 day before treatment with Etoposide with and without different inhibitors. Cells were counted after 4 days of treatment, reseeded into fresh medium, and counted again after an additional 2 days.

To determine clonogenic capacity, cells were treated with Etoposide with and without different inhibitors. After 4 days, 5000 cells were seeded into fresh medium in quadruplicates in 6‐well plates. After an additional 7 days, the samples were fixed with 10% formaline and stained with 0.5% (w/v) crystal violet before the number of colonies was assessed.

### Kinase assay

Asynchronously growing RPE Cyclin A2‐eYFP cells or RPE cells released for 6 h from thymidine block (2.5 mm, 24 h) were treated with DMSO or with indicated doses of Etoposide or Neocarzinostatin for 4 h and lysed in ice‐cold IP buffer (50 mm HEPES pH 7.4, 150 mm NaCl, 10% glycerol, 0.1% NP‐40) supplemented with protease and phosphatase inhibitors (Roche). Normalized cell extracts were incubated with 2 μg of control IgG, antibody against GFP or Cdk2 for 1 h and for additional 1 h with protein A/G beads (Pierce). Beads were washed twice with IP buffer and once with kinase buffer (25 mm MOPS pH 7.2, 12.5 mm glycerol 2‐phosphate, 25 mm MgCl_2_, 5 mm EGTA, 2 mm EDTA, and 0.25 mm DTT). Beads were incubated with kinase buffer supplemented with 100 μm ATP, 5 μCi ^32^P‐γ‐ATP and purified GST‐Cdk substrate (2 μg) for 20 min at 30 °C. Where indicated, 12 μm Roscovitine was added to the kinase buffer. Reaction was stopped by addition of 4× Laemli buffer and boiling for 5 min. Proteins were separated by SDS–PAGE and phosphorylation of GST‐Cdk substrate was detected by autoradiography. Alternatively, RPE Cyclin B1‐eYFP cells were synchronized by thymidine for 24 h, released for 6 h when cells reach G2 phase and then treated with Etoposide for 4 h. Immunoprecipitation and kinase assay were performed as mentioned above, except GST‐LAMS22 substrate was used. Phosphorylation of GST‐LAMS22 was detected by immunoblotting using an antibody against Lamin A/C phosphorylated at Ser22 (see above).

### Cell viability and cell size

RPE cells were seeded at 10 000 per well to 6‐well plates 1 day before treatment with Etoposide (1 or 2 μm). Where indicated, Roscovitine (25 μm) was added to cells 1 h after Etoposide treatment. Cells were harvested and analyzed 4 days after treatment. To evaluate cell viability, cells were stained with Annexin‐V‐FITC following manufacturers recommendations (Life Technologies, Carlsbad, CA, USA). Hoechst 33258 was added 5 min before measurement. Cells were analyzed using BD LSRII flow cytometer and FlowJo analysis software. Living cells were gated as nondebris Annexin‐V negative Hoechst negative. Cell size was determined as FSC area in living cells.

### Metabolic activity

RPE cells were seeded at 800 cells per well to 96‐well plate 1 day before treatment with Etoposide (1 or 2 μm). Where indicated, Roscovitine (25 μm) was added to cells 1 h after Etoposide treatment. Cell proliferation was measured 4 days after treatment by addition of resazurin (30 μg mL^−1^) to growth media and fluorescence signal (excitation wavelength 560 nm, emission wavelength 590 nm) was measured after 1 h using EnVision plate reader (PerkinElmer, Waltham, MA, USA).

### Cell cycle

RPE cells were seeded at 120 000 per well to 6‐well plates 1 day before treatment with Etoposide (1 or 2 μm). Where indicated, Roscovitine (25 μm) was added to cells 1 h after Etoposide treatment. Cells were harvested and fixed 4–24 h after Etoposide treatment. After permeabilization, cells were stained with DAPI to evaluate cell cycle profile. Samples were analyzed using BD LSRII flow cytometer and FlowJo analysis software, FlowJo, Ashland, Oregon, USA.

### Statistical analysis

Experiments were performed at least three times unless stated otherwise. Statistical hypothesis testing for differences between the means of two populations was carried out using Welch's *t*‐test, an adaptation of Student's *t*‐test, that is reliable for populations with unequal variances and sample sizes, and remains robust for skewed distributions (Fagerland, 2012).

Statistical hypothesis testing for observed differences in sets of categorical data was carried out using Pearson's χ^2^ test.

## Funding

This work was supported by grants from the Swedish Research Council, the Swedish Foundation for Strategic Research, and the Swedish Cancer Society. LM was supported by the Grant Agency of the Czech Republic (13‐18392S) and by RVO: 68378050.

## Author contributions

E.M. and A.L. designed the study and wrote the manuscript. E.M., H.SC., K.B., and L.M. carried out the experiments. E.M., H.SC., K.B., L.M., and A.L. analyzed the data.

## Conflict of interest

The authors declare that they have no conflict of interest.

## Supporting information


**Fig. S1** Cdk1 and Cdk2 activity, but not Plk1 regulates Cyclin B1 nuclear accumulation upon DNA damage.
**Fig. S2** Cdk activity induces senescence upon DNA damage.
**Fig. S3** Cdk activity is retained after DNA damage in RPE and U2OS cells.
**Fig. S4** Cdk activity promotes p21 production in RPE and U2OS cells.
**Fig. S5** Cdk activity promotes p21 production in RPE and U2OS cells.
