# Supplementary material for: Residual Cdk1/2 activity after DNA damage promotes senescence
Source: Aging Cell. 2017 Mar 26;16(3):575–84. doi: 10.1111/acel.12588 (PMC5418196; doi:10.1111/acel.12588)

Supplementary Figure 1

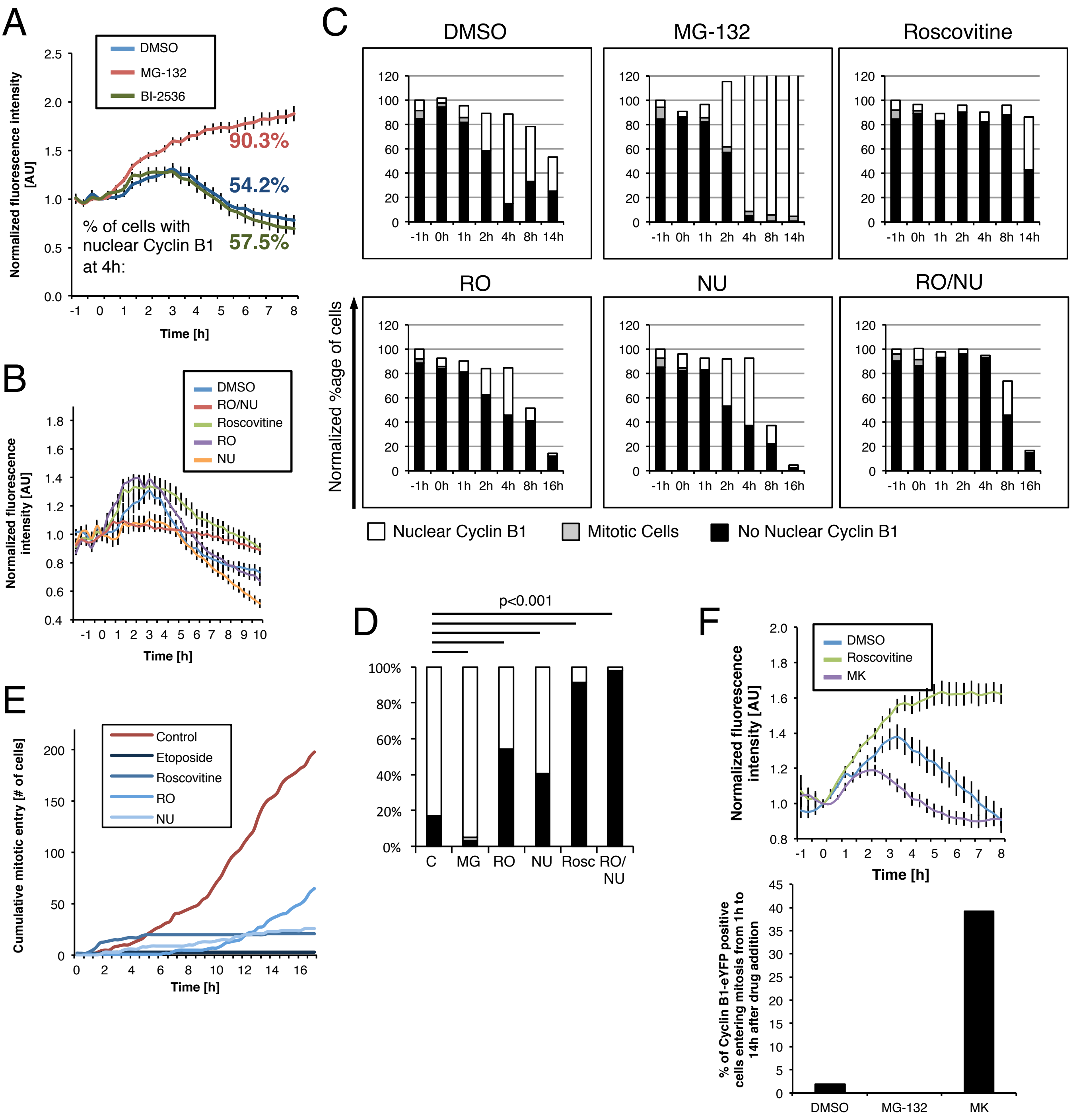

Supplementary Figure 2

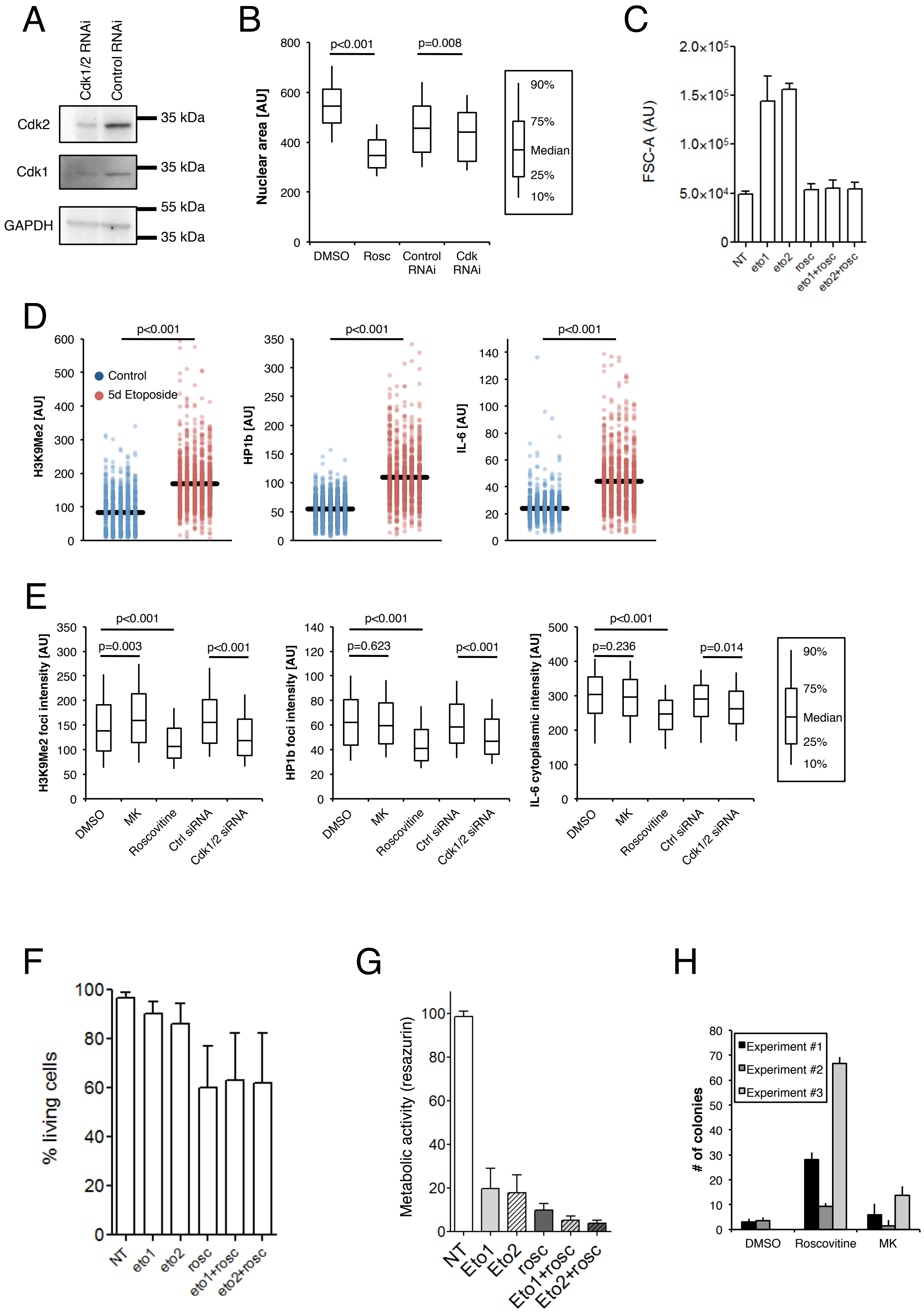

Supplementary Figure 3

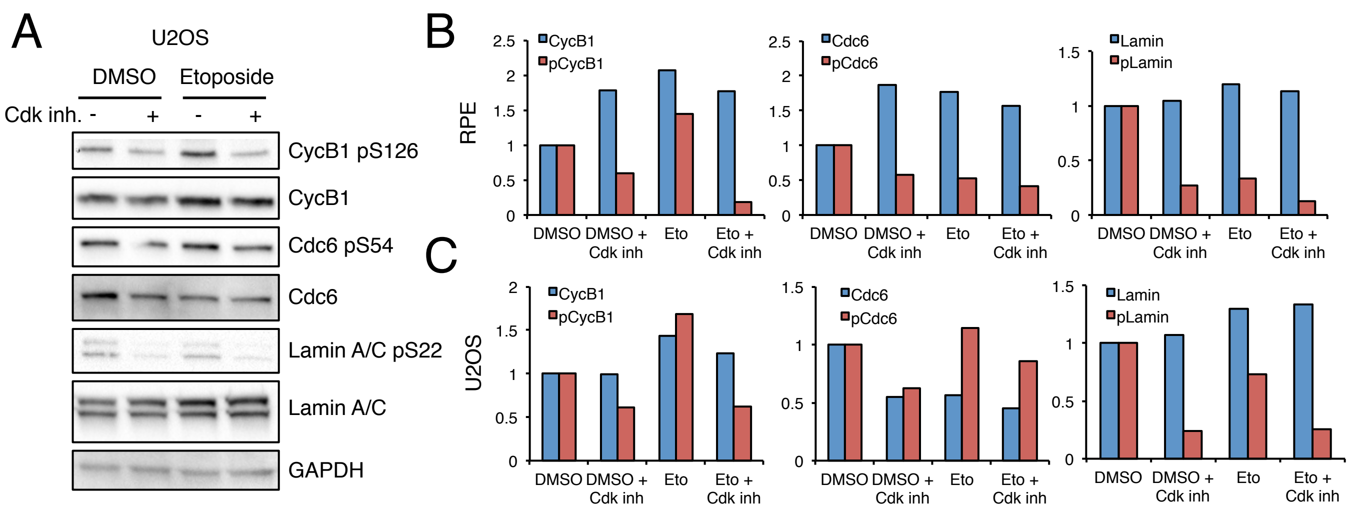

D

	Ratio phosphorylation/total	
**Lamin vs pLamin**	RPE	U2OS
DMSO	1	1
DMSO + Cdk inh	0.26	0.22
Eto	0.28	0.56
Eto + Cdk inh	0.11	0.19
**CycB1 vs pCycB1**	RPE	U2OS
DMSO	1	1
DMSO + Cdk inh	0.33	0.61
Eto	0.69	1.17
Eto + Cdk inh	0.10	0.50
**Cdc6 vs pCdc6**	RPE	U2OS
DMSO	1	1
DMSO + Cdk inh	0.31	1.12
Eto	0.29	2.03
Eto + Cdk inh	0.26	1.89

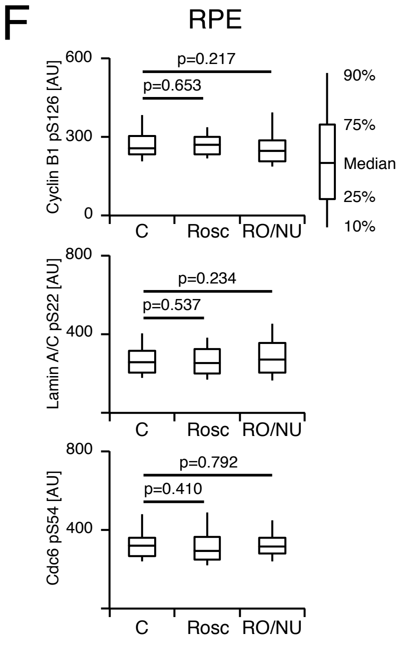

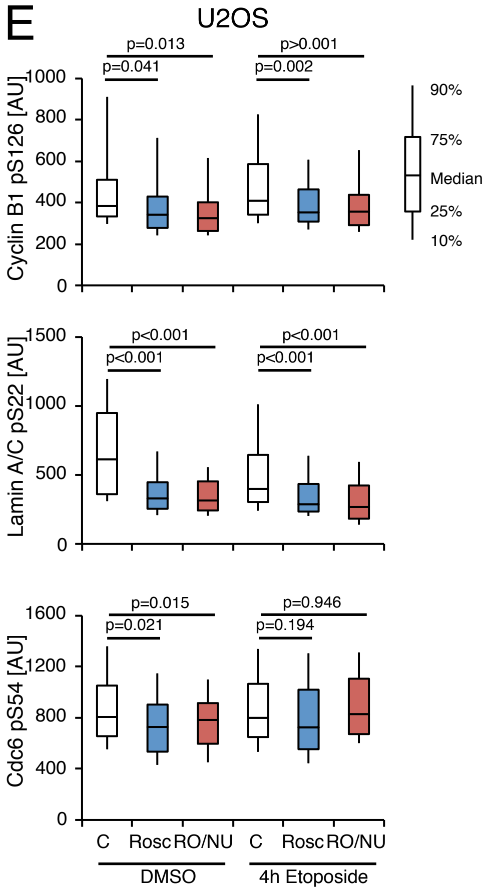

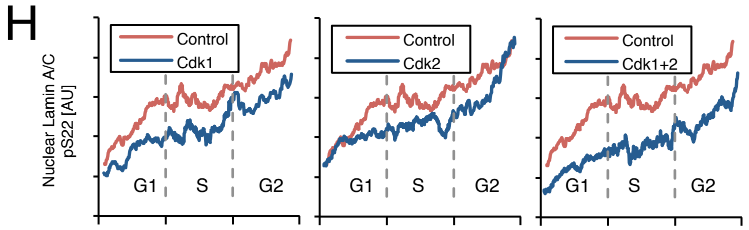

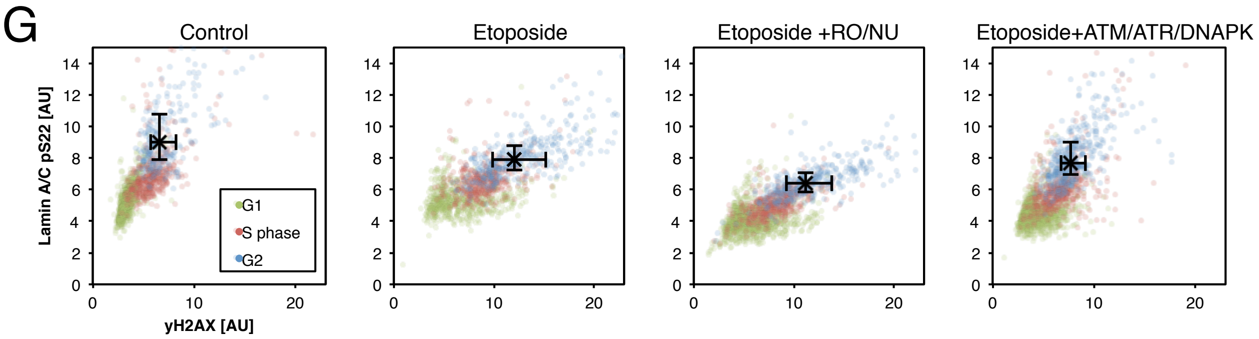

Supplementary Figure 3

I

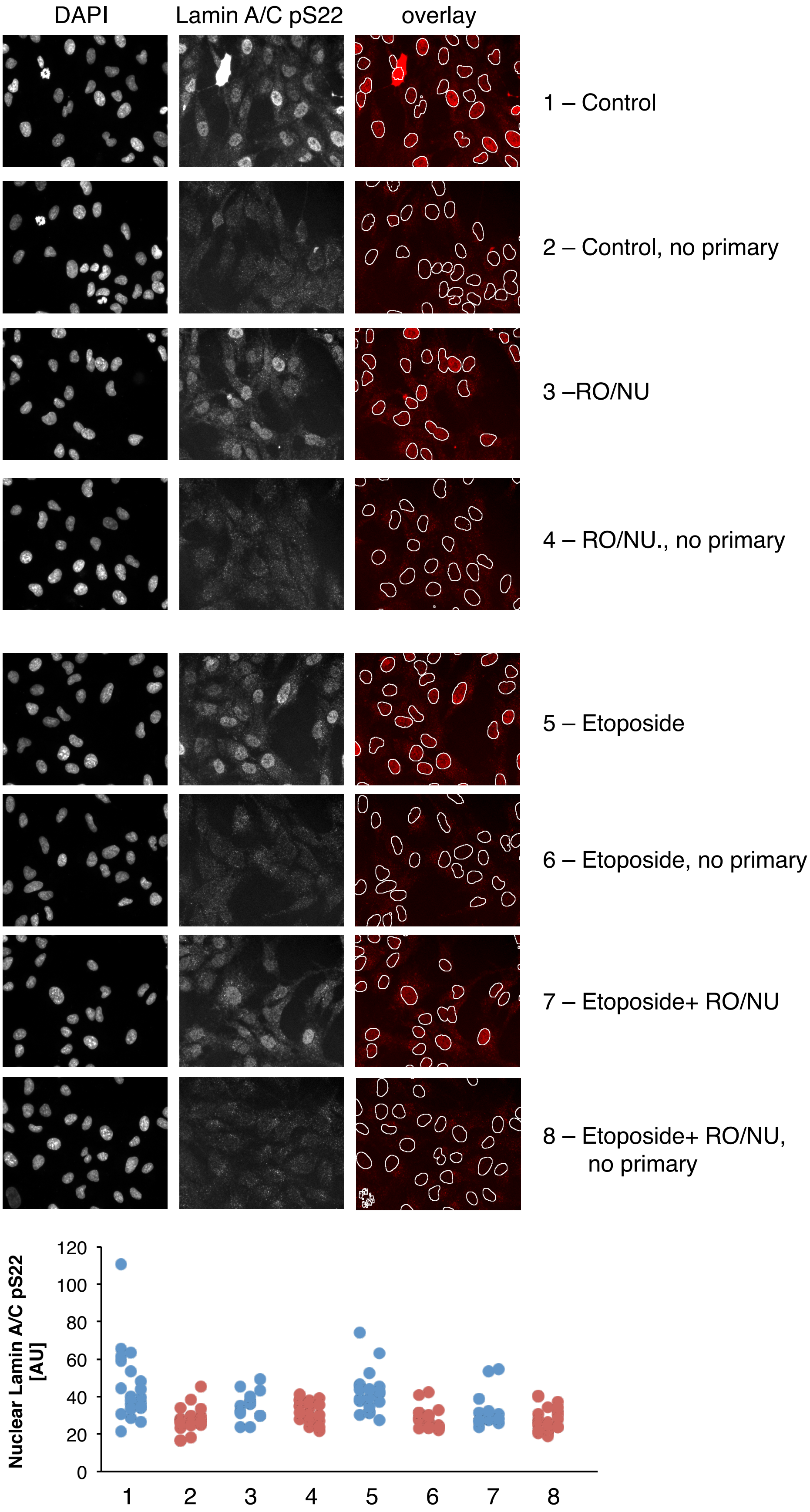

Supplementary Figure 4

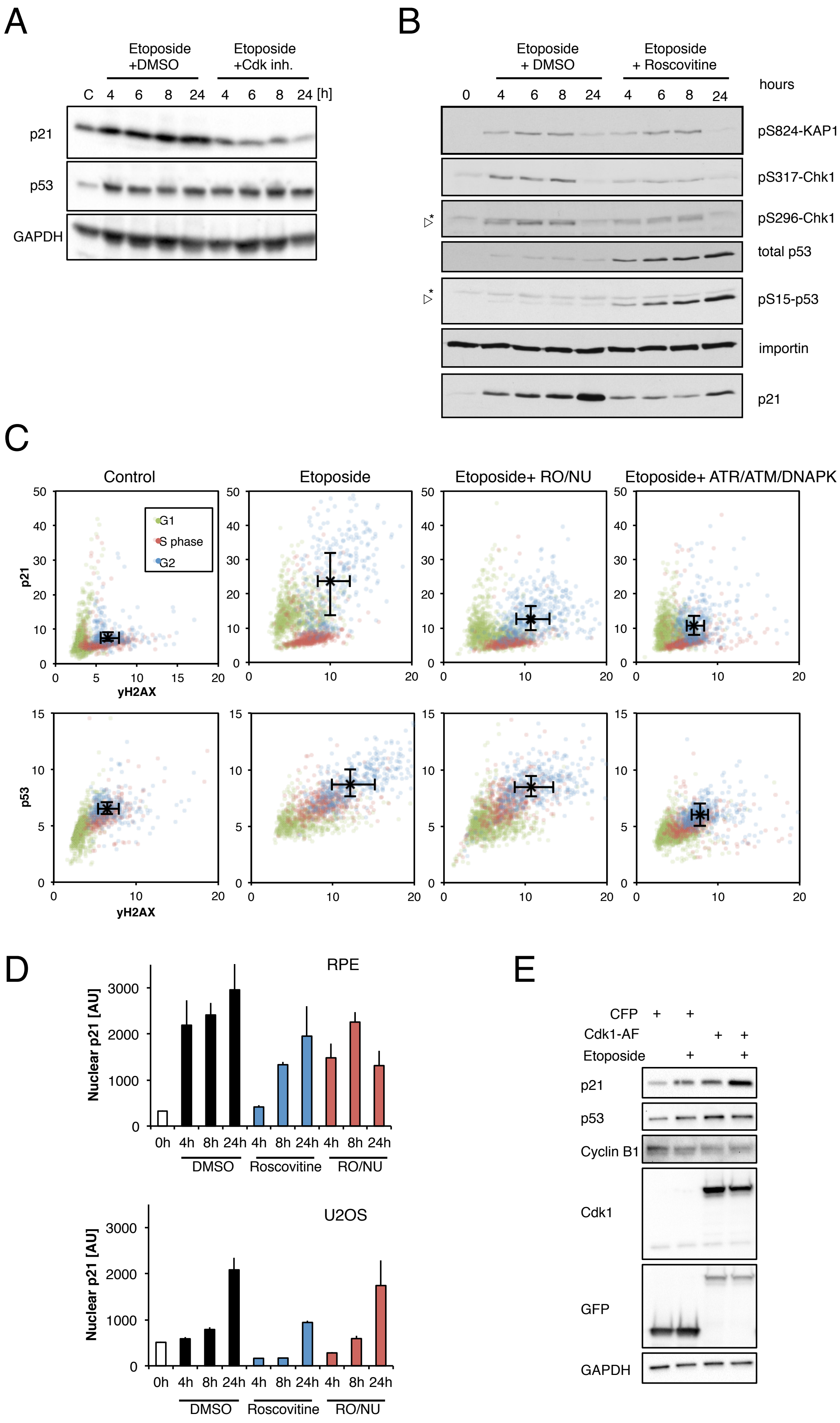

Supplementary Figure 5

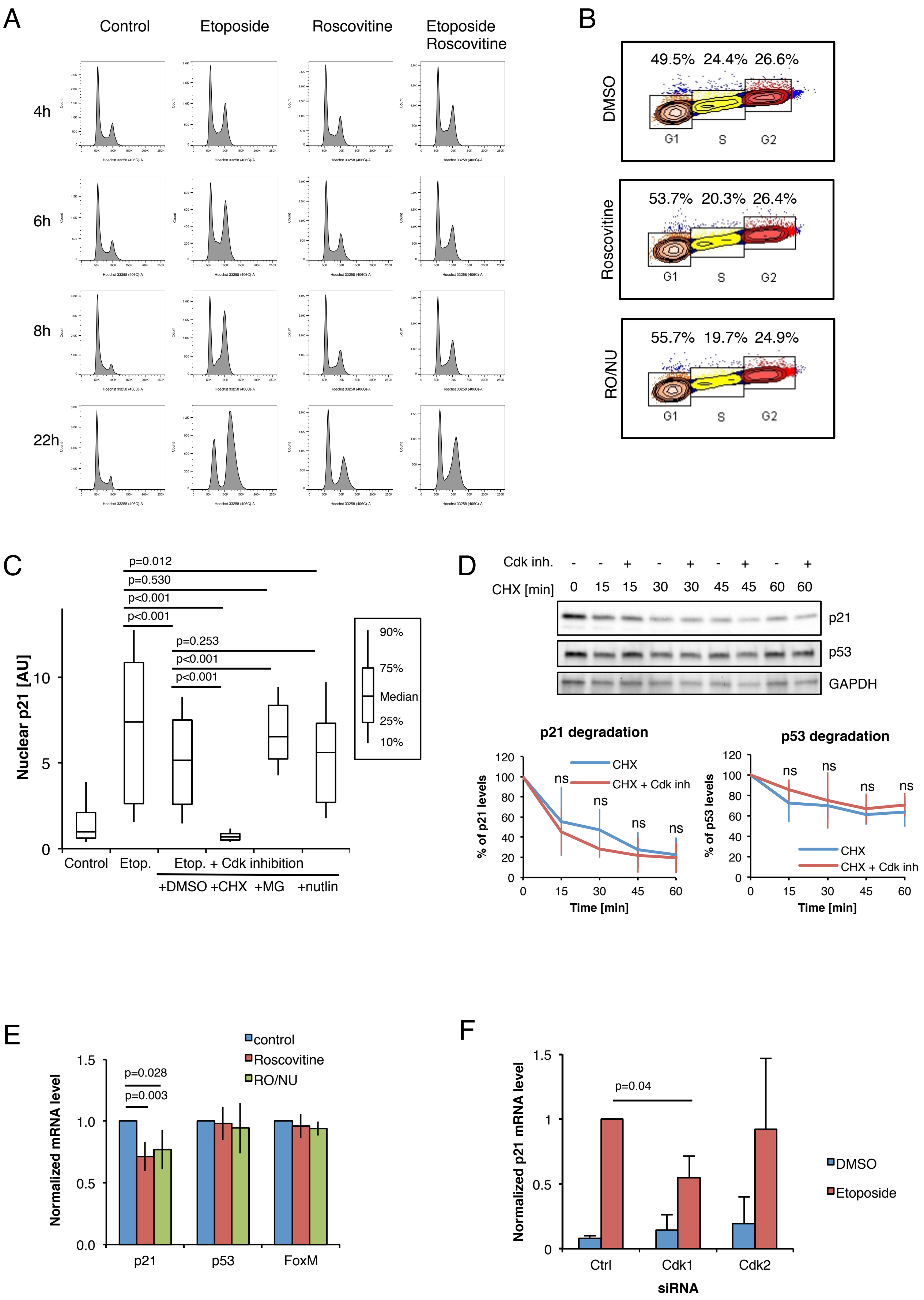

Supplement: Supplementary file 1 — Fig. S1 Cdk1 and Cdk2 activity, but not Plk1 regulates Cyclin B1 nuclear accumulation upon DNA damage. Fig. S2 Cdk activity induces senescence upon DNA damage. Fig. S3 Cdk activity is retained after DNA damage in RPE and U2OS cells. Fig. S4 Cdk activity promotes p21 production in RPE and U2OS cells. Fig. S5 Cdk activity promotes p21 production in RPE and U2OS cells. [file ACEL-16-575-s001.pdf]
